# Quantitative Bone Assessment in Medication-Related Osteonecrosis of the Jaw Using Fractal Analysis: A Systematic Review of the Literature and Clinical Perspectives

**DOI:** 10.3390/dj14040207

**Published:** 2026-04-02

**Authors:** Aleksandra Misiejuk, Paulina Adamska, Agata Żółtowska, Adam Zedler

**Affiliations:** 1 Division of Oral Surgery and Implantology, Faculty of Medicine, Medical Univeristy of Gdańsk, 80-210 Gdańsk, Poland; aleksandra.misiejuk@gumed.edu.pl (A.M.); adam.zedler@gumed.edu.pl (A.Z.); 2 Division of Conservative Dentistry, Faculty of Medicine, Medical Univeristy of Gdańsk, 80-210 Gdańsk, Poland

**Keywords:** BRONJ, bisphosphonate-related osteonecrosis of the jaw, fractals, mathematical concepts, osteonecrosis, radiology

## Abstract

**Background**: Contemporary dentistry increasingly relies on tools and methods derived from the exact sciences, particularly mathematics and physics, to better understand the complexity of biological processes. One such tool is fractal analysis (FA), which enables the characterization and quantification of irregular, complex, self-similar structures commonly observed in nature in the form of the fractal dimension (FD). In oral radiology, it has been found useful for describing structural changes in bone tissue. **Objective**: The aim of this review is to present the current state of knowledge regarding the application of fractal analysis in the management of patients with, or at risk for, medication-related osteonecrosis of the jaw (MRONJ), with particular emphasis on its diagnostic and prognostic potential. This paper summarizes key research findings, and discusses the principal challenges and limitations associated with the use of this method of analysis in MRONJ cases. **Materials and Methods**: The inclusion criteria were as follows: original papers, the presence of MRONJ, and fractal analysis. In order to find relevant studies, international databases, including PubMed and Google Scholar, were searched. The last search was performed on 29 November 2025. Six articles were included in the systematic review. **Results**: The majority of the review studies show lower FD values for MRONJ patients and healthy control groups. The values are the lowest for necrotic lesions and highest for perinecrotic bone tissue. **Conclusions**: FD values calculated from radiological images of the jaws can be used to differentiate healthy and MRONJ-affected patients and to describe necrotic lesions. Fractal analysis has potential to be used in the diagnosis and monitoring of MRONJ after further studies and standardization of methodology.

## 1. Introduction

Contemporary medicine increasingly relies on tools and methods derived from the exact sciences, particularly mathematics and physics, to better understand the complexity of biological processes. Natural environments frequently exhibit complex and irregular structures that fall outside the framework of classical Euclidean geometry. The shapes of clouds, shorelines, trees, and tissues like bones and lungs all show the property of self-similarity, meaning that their appearance remains consistent irrespective of the scale of magnification applied. Mandelbrot [1], in his work, uses the term “fractal” from the Latin ‘fractus’, meaning ‘broken’ or ’fragmented’, to describe such structures. A fractal set contains self-similar elements, which are independent of scale, and the fractal dimension (FD) represents the amount of space filled by a fractal set [1]. The FD serves as a quantitative parameter utilized for the measurement of the complexity inherent in the examined objects and can be used to describe radiographs. Multiple methods of fractal analysis (FA) on grey-scale images exist, each with its own limitations. Natural images, including ones of bone tissues, are usually not ideal fractal sets; hence, the computed FD depends on the applied method [2].

For the characterization of bone tissue in radiological images, the box-counting method is the most frequently used technique. In multiple studies presented in this article, researchers have tried using this method to describe lesions in bones affected by osteonecrosis. The FD is calculated on radiological images in selected ROIs (regions of interest) [3].

Osteonecrosis of the jaw (ONJ) is caused by inadequate blood supply. The bone does not receive enough oxygen and nutrients, which leads to creating areas of necrotic bone. Symptoms of ONJ include pain, swelling and infection of soft tissues, fistulas, loosening of teeth, drainage, and a feeling of heaviness or numbness in the jaw [4,5,6,7,8]. Osteonecrosis can result from trauma, inadequate fit of prosthetic restorations or fillings, radiotherapy, the use of antiresorptive or antiangiogenic drugs, inflammation, and systemic factors such as metabolic diseases or smoking [8]. Given the focus of this article, the authors concentrated on drug-related osteonecrosis. The first cases of osteonecrosis of the jaw—especially bisphosphonate-related osteonecrosis of the jaw (BRONJ)—were reported back in 2003 [5]. Bisphosphonates are antiresorptive drugs (ADs) used for managing cancer-related conditions, like bone metastasis. They are also used for the prevention of osteoporosis-related fractures (fragility fractures) and treatment of other diseases such as osteogenesis imperfecta, Paget’s disease, multiple myeloma, and malignancy-induced hypercalcemia. The beneficial effects of antiresorptive drugs mainly rely on their capacity to reduce the bone turnover rate, making the bone more fracture-resistant. Bisphosphonates reduce bone resorption by inhibiting osteoclasts’ function and inducing their apoptosis by inhibiting farnesyl pyrophosphate synthase, an enzyme in the HMG-CoA reductase pathway [6]. Due to the introduction of other pharmacologic agents producing similar effects (denosumab, romosozumab), the currently preferred term is medication-related osteonecrosis of the jaw (MRONJ) [7,8]. The case definition of MRONJ includes all the following elements:

1. Current or previous treatment with antiresorptive therapy alone or in combination with immune modulators or antiangiogenic medications.
2. Exposed bone or bone that can be probed through an intraoral or extraoral fistula(e) in the maxillofacial region that has persisted for more than eight weeks.
3. No history of radiation therapy to the jaws or metastatic disease to the jaws [7].

**Figure 1:**
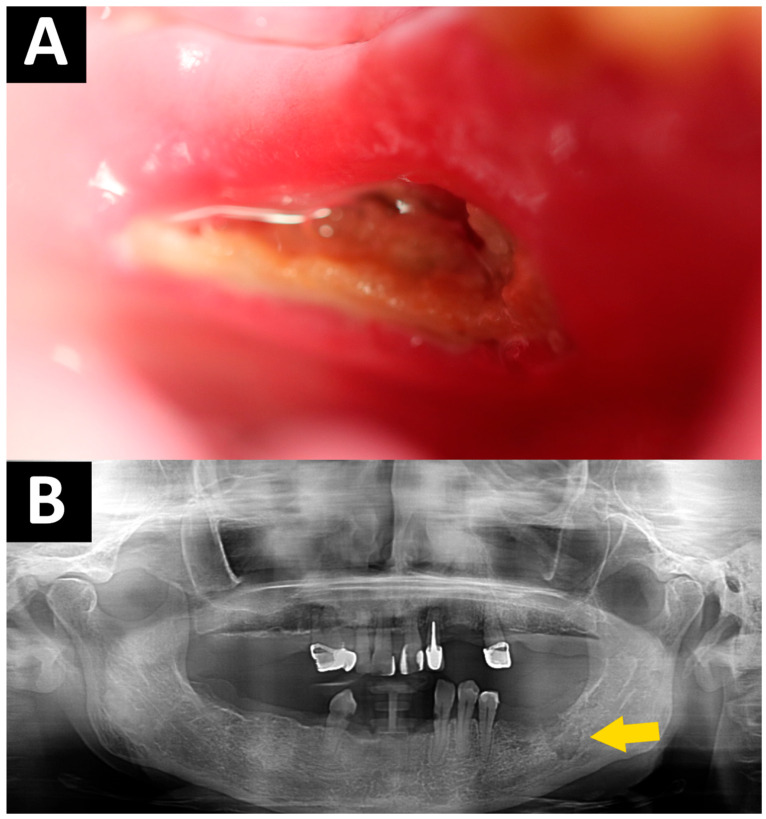
MRONJ—the same patient: (**A**) Clinical manifestation of MRONJ. (**B**) Orthopantomography—osteolysis of bone—yellow arrow.

The most commonly used MRONJ classification and treatment strategies are the guidelines of the American Association of Oral and Maxillofacial Surgeons (AAOMS). They focus on clinical staging based on symptoms, bone exposure, and the presence of infection, with these staging strategies guiding general treatment recommendations (conservative and surgical). Diagnosis is difficult. In stage 0, there are no clinical symptoms, only radiological findings. Radiographic findings reveal a radiolucent area (osteolytic ring) around the nucleus, which forms a sequestrum (Figure 1, Table 1). Therefore, regular clinical and radiological follow-ups of patients at risk of MRONJ are very important [7,8,9,10,11,12,13].

The aim of this review is to present the current state of knowledge regarding the application of fractal analysis in the management of patients with, or at risk of, MRONJ, with particular emphasis on its diagnostic and prognostic potential. This paper summarizes key research findings, and discusses the principal challenges and limitations associated with use of this method of analysis in MRONJ cases.

## 2. Materials and Methods

The PRISMA (Preferred Reporting Items for Systematic Reviews and Meta-Analyses) guidelines were used (Supplementary Material Table S1) [9]. The study protocol was registered with the PROSPERO (Prospective Register of Systematic Reviews, CRD420251242539). The research questions were as follows: Can fractal analysis be important in the diagnosis of medication-related osteonecrosis of the jaw? Does the value of the fractal dimension (FD) measured on a radiographic image reflect the presence and progression of MRONJ? What are the most reliable imaging modalities (orthopantomography (OPG), cone-beam computed tomography (CBCT), or regions of interest (ROIs)) for measuring the fractal dimension in the monitoring of MRONJ? The PICO (Population, Intervention, Comparison, Outcome) framework was applied as follows:

- Population (P): a study with a min. of 10 patients with medication-related osteonecrosis of the jaw (MRONJ criteria: (I) current or previous treatment with antiresorptive therapy alone or in combination with immune modulators or antiangiogenic medications; (II) exposed bone or bone that can be probed through an intraoral or extraoral fistula(e) in the maxillofacial region that has persisted for more than eight weeks; (III) no history of radiation therapy to the jaws or metastatic disease to the jaws [7]).
- Intervention (I): fractal analysis performed on a radiological image of the mandible.
- Comparison (C): conventional clinical and radiological evaluation.
- Outcome (O): a simplified, faster diagnostic process.

### 2.1. Search Strategy

The search was conducted using the PubMed, Google Scholar, Scopus, and Web of Science databases. The search terms used were ‘osteonecrosis of the jaw’, ‘bisphosphonates-related osteonecrosis of the jaw’, ‘BRONJ’, ‘medication-related osteonecrosis of the jaw’, ‘MRONJ’, ‘fractals’, and ‘fractals analysis’. The last manuscript search was conducted on 29 November 2025. The search was not limited by the time of publishing.

### 2.2. Selection Criteria

#### 2.2.1. Inclusion Criteria

The inclusion criteria were as follows: original papers, presence of MRONJ, performed fractal analysis, and studies published in English.

#### 2.2.2. Exclusion Criteria

The exclusion criteria were as follows: review articles, case reports, pre-prints or commentaries, articles about osteonecrosis of the jaw not related to medications, studies that did not perform fractal analysis, and studies written in languages other than English.

### 2.3. Data Screening

The initial screening was conducted by the first author through a title-based assessment to identify studies meeting the inclusion criteria. A subsequent critical appraisal of the selected articles was performed by the first author under the supervision of the author A.Ż. The second author, P.A., supervised the entire search strategy, screening, and data extraction process and served as the final arbiter in cases of disagreement. Duplicate records were removed, and the remaining articles were further assessed for eligibility based on abstract review. Full-text versions of potentially relevant studies were then retrieved and evaluated according to predefined criteria. Ultimately, seven publications met the criteria and were included in the review.

### 2.4. Data Extraction

After completing the search and screening process, data from all articles selected for inclusion were analyzed. The findings from each study were extracted and summarized in a table. In addition, key information—such as the first author, year of publication, country of research, procedure performed, number of patients, use of fractal analysis, and main conclusions—were collected and compiled into an overview table to provide a general summary of all included studies.

### 2.5. Quality Assessment

A formal assessment of publication bias was not performed, as such analyses are generally considered unreliable when fewer than 10 studies are included. However, to assess the methodological quality and risk of bias of the included studies, we performed a quality assessment using the Newcastle–Ottawa Scale (NOS). Study quality was appraised using the NOS, which assesses three core domains: the selection of studies, comparability between groups, and exposure evaluation. The assessment was performed by the second author, and any disagreements were resolved through discussion to reach consensus. The NOS assigns up to a maximum of nine stars across the three domains, with studies receiving a score of seven or higher classified as high-quality.

## 3. Results

### 3.1. Study Characteristic

**Figure 2:**
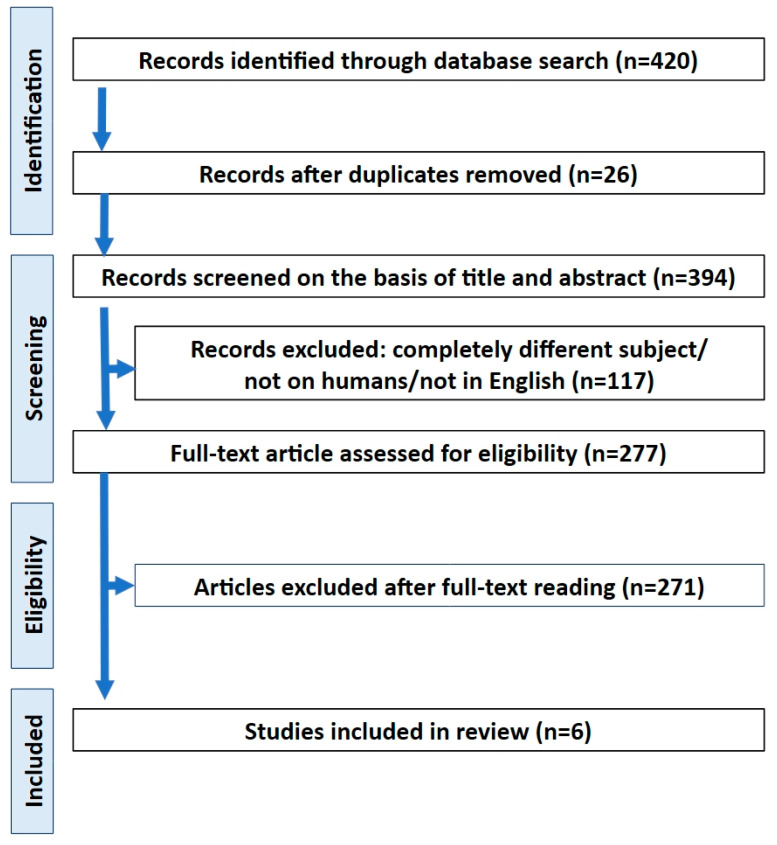
PRISMA flow diagram depicting the process followed for the selection of the studies.

Of the 420 articles initially identified, six met the inclusion criteria. Three studies assessed the fractal dimension using OPG, and two employed CBCT, while one study applied and compared both imaging modalities. Collectively, the included studies comprised a total of 232 patients. The study selection process is illustrated in the PRISMA flow diagram (Figure 2). An overview of the selected publications is provided in Table 2.

Torres et al. [10] conducted a CBCT-based study of patients affected by BRONJ. As a result, they calculated that the odds of being a BRONJ patient vs. being a control were considerably higher for individuals with higher FD scores measured at an ROI located above the mental foramen (in the axial plane). Areas without visible bone exposure also showed differences in FD values, which suggest that the FD might be a useful tool for the detection of early bone alterations that precede clinical symptoms. Conversely to the other studies reviewed, the calculated FDs of patients with osteonecrosis were higher than the control group [10].

Sahin et al. [11] compared two groups of MRONJ-affected patients, group I at stage 0 (without bone exposure) and group II with more advanced disease (stages 1, 2, 3). Significant differences were found only in 1 out of 4 selected ROIs: in the molar region, superior to the mandibular canal, on the distal side of the mental foramen. In a comparison of the radiological findings, focal and diffuse sclerosis, sequestrum, and enhancement of the inferior alveolar canal (IAC) were significantly more frequent in group II than in group 1, and all of them occurred more frequently in the mandible than in the maxilla [11]. A comparison of the FD values for an ROI containing cancellous bone above the mandibular canal is provided in Table 3.

Two studies compared the FD values of healthy patients and those affected by MRONJ. Aslan et al. [15] calculated the FD from both CBCT and PR images, while Bachtler et al. [12] used only CBCT scans. In the first study, the FD value in the MRONJ study group (1.684 ± 0.051) was approximately 3.5% lower than that in the control group (1.745 ± 0.026) and was statistically significant. It was also noted that the groups were not matched in age [12].

Aslan et al. [15] also proved that the FD decreased for MRONJ-affected bone in both OPG and CBCT images. In contradiction to Bachtler’s study [12], only in OPG images was the difference significant in regard to the discrimination of healthy and MRONJ-affected bone. An ROI that contained trabecular bone located distal to the mental foramen, above the IAC, yielded the most reproducible results. Apart from the FD, the authors also measured other quantitative radiomorphometric parameters. They verified that the FD, lacunarity, mean grey value (MGV), bone area fraction (BA/TA), and trabecular separation (Tb.Sp) could reliably differentiate MRONJ-affected bone in OPG images but only mandibular cortical thickness (MCT) could reliably differentiate MRONJ-affected bone in both OPG and CBCT [15].

Panneer-Selvam N. et al. [14] explored the possible benefits of drug holidays for patients taking BP and denosumab with research based on the lesions in the trabecular bone structure. Out of 18 patients included in the study, 8 developed MRONJ despite being on drug holiday (44.44%). There was no significant difference in the FD values between drug holiday and pre-drug holiday groups, which implies that there were no substantial alterations in the trabecular bone pattern. This evidence questions the advantage of a drug holiday, especially considering the long-term drug reservoir effect [14].

Schulz et al. [16] studied osteoradionecrosis and medication-related osteonecrosis in separate groups. The study compared bone-formation-related markers—sclerostin and alkaline phosphatase (ALP)—with radiographic findings in search of correlation. ROIs were selected not by the anatomical location but by the state of the bone, including: a necrotic site, perinecrotic bone, and a non-lesional-bone site on the contralateral side. Sclerostin levels were significantly elevated in the ORJ and MRONJ groups. The ALP level was higher in the control group than in the ORJ and MRONJ groups. Statistically significant differences were observed across all evaluated radiological parameters: the MGV, standard deviation grey value (SDgv), FD, and lacunarity. The FD values were the lowest for the necrotic sites (median 1.21), and for perinecrotic bone, the FD was slightly higher (median 1.28) than the values of non-affected bone (1.27) with *p* < 0.05. The study utilized the revised, simplified version of White and Rudolph’s [17] original method for calculating the FD, but it was proved that they exhibited good agreement, with a significant positive correlation in values [16,18].

### 3.2. Risk of Bias

Four of the selected studies were considered to be of good quality, one moderate, and two were low-quality. The risk-of-bias assessment using the NOS is described in Table 4.

## 4. Discussion

All of the studies included in this review employed the box-counting method for FD analysis, utilizing the protocol originally described by White and Rudolph [17] or a variation of this method. New approaches have made calculations simpler, faster, and possibly more accurate but can add to discrepancies between the studies [19,20]. The box-counting method has been experimentally applied in dentistry among others to evaluate bone quality in implant osseointegration and to detect lesions in periodontal tissues [20,21]. Consequently, there is substantial interest in exploring the clinical utility of FD analysis for other conditions affecting the bones.

In MRONJ, radiographic findings can precede the clinical manifestation. Diagnostic imaging features associated with MRONJ described by the AAOMS include: alveolar bone loss or resorption not attributable to chronic periodontal disease; lesions in trabecular pattern sclerotic bone and no new bone in extraction sockets; regions of osteosclerosis involving the alveolar bone and/or the surrounding basilar bone; and thickening/obscuring of the periodontal ligament (PDL)—thickening of the lamina dura, sclerosis, and decreased size of the periodontal ligament space [7].

The use of bone exposure as a criterion in the diagnosis of MRONJ causes delays in diagnosis and resistance to treatment. Stage 0 (Table 1) presents nonspecific clinical findings, radiographic lesions, and symptoms. A lack of specific clinical features often causes diagnostic difficulty for clinicians. In such cases, radiologic examination may be useful for the correct diagnosis and evaluation of osseous lesions [11].

At the same time, the AAOMS remains concerned that overemphasizing variable radiographic features often attributed to MRONJ may overestimate the true disease frequency by including false positives in the numerator (e.g., cases with radiographic findings suggestive of MRONJ), but these patients do not fit the criteria for the diagnosis of MRONJ [7].

Since FA provides an objective, quantitative method for bone assessment on radiological images, it could provide the necessary help in improving the diagnostic process. The reviewed literature presents conflicting evidence regarding the utility of cone-beam computed tomography in conducting fractal analysis. In the study of Aslan et al. [15], the authors conclude that CBCT is not a reliable imaging modality for FA calculations, while Torres et al. [10] and Bachtler et al. [12] performed FA from CBCT with satisfying results. Several factors could contribute to this difference. In the studies of Bachtler et al. [12] and Aslan et al. [15], the groups were not matched in age. Both studies selected patients for control groups (Bachtler et al.: mean age 57.6 ± 12.2; Aslan et al.: mean age 57.95 ± 8.27) significantly younger than the study groups (Bachtler et al.: mean age 76.5 ± 9.4 years; Aslan et al.: mean age 68.12 ± 11.2) [10,12,15].

Different drug dispositions could also have affected the results of the mentioned studies. One article does not specify the drugs that were administered to the patients [12], and in most of the studies, patients taking Mo and BP were assigned to the same study groups [11,14,16]. Bisphosphonates can be administered orally or intravenously. They create a reservoir in bone without being metabolized. Even after treatment, they are gradually released over months or years. In contrast, denosumab, a monoclonal antibody administered via subcutaneous injection, is metabolized in the body and does not bind to bone. Denosumab’s effects on bone remodelling are mostly diminished within six months after the last dose. These pharmacokinetic differences lead to varying impacts on jawbones. Bisphosphonates are known to accumulate primarily in trabecular bone and increase bone density in these regions, while denosumab has a more pronounced effect on the cortical bone, with a limited influence on trabecular bone [6,7,22].

None of the studies differentiated patients by the acquired cumulative dose of the medication. The risk of developing MRONJ is proportional to the duration of therapy and cumulative dose, and also influenced by the route of administration and type of BP. This suggests that lesions of the morphology of the bone may also proportionately vary in severity. More than 95% of cases reported in the literature have occurred in patients with metastatic bone disease after receiving long-term, high-dose, *i.v.* BP. Patients in the studies also suffered from different diseases that were treated with ADs. Patients treated for oncologic conditions were assigned to the same study groups as those treated for osteoporosis. The dose of the medication used in oncology is about 1012 times the dose used for osteoporosis [23]. In patients who were undergoing the therapy but did not experience MRONJ, the type of administered drug, type of systemic disease, and time of the therapy influenced the FD values differently [24].

The reviewed studies consistently show (Table 3) that FD values calculated from radiological images of jaws can be used to differentiate between healthy and MRONJ patients. A decrease in FD values theoretically correlates with the trabecular bone structure becoming coarser and less complex. Since osteonecrosis involves bone degeneration, lower FD values would be expected in MRONJ patients due to the presence of necrotic lesions. Interestingly, in one study [10], the authors observed a counterintuitive trend of higher FD values in the study group.

A key point of variation among studies is the method used for selecting ROIs. While it has been demonstrated that fractal dimensions determined from ROIs of digital radiographic images of alveolar bone are relatively insensitive to small variations in X-ray exposure, beam alignment, and ROI position, other methodological differences remain. For instance, some studies selected ROIs based solely on anatomical criteria, irrespective of whether the bone in that region was necrotic or not [10,11,12,15]. The most appropriate site of the mandible for evaluating the FD on panoramic radiographs was the region above the mandibular canal distal to the mental foramen [11,15]. For CBCT, it was above the mandibular canal in the axial plane (Table 3) [10]. Others focused on the state of the bone, choosing ROIs by the location corresponding to the necrotic lesions [16]. This approach revealed that, while the FD decreased significantly at the lesion site, the perilesional area presented FD values that were even higher than those of healthy bone. This phenomenon could be explained by the adaptive response of the adjacent bone. The authors highlight that this finding could also be considered in cases where the lesion is not yet clinically visible.

In terms of assessing the risk of MRONJ in patients undergoing antiresorptive treatment, contradictory findings exist in the recent literature regarding lesions in terms of FD values caused by the medication. Some authors report no significant differences in FD between patients taking BP and control groups [25,26,27]. Conversely, other studies describe significantly lower FD values in patients receiving antiresorptive treatment compared to control groups [24,28]. These latter findings further specify that the type of administered drug, route of administration, and systemic disease (oncologic or osteoporosis) differentially influence FD values across selected ROIs.

In the study of Kurşun et al. [29], the FD was used to compare changes in trabecular bone structure before and after a year of using a complete removable denture, removable partial denture, and partial fixed prosthesis in patients taking BP and a control group. Complete and partial removable dentures were linked to a decrease in FD value in the maxilla and mandible in both the study and control group, but in the control group, the decrease was significantly greater (in the molar region). An increase in FD values over time was obtained only in the BP-taking group with partial fixed maxillary and mandibular prostheses [29]. These specific observations suggest that FD analysis is a valuable tool for describing very precise, characteristic changes in trabecular bone structure, which holds promise for creating diagnostic and risk-assessment tools for MRONJ in the future. In comparison to conventional clinical and evaluation, the FD was proved to detect lesions in bone structure even without the clinical manifestation of bone exposure [10,11].

It is necessary to consider combining the FD with other radiomorphometric parameters such as the mandibular cortical thickness (MCT), mandibular cortical width (MCW), lacunarity, mean grey value (MGV), and panoramic mandibular index (PMI). These parameters can be measured on the same radiographic images as the FD and have been proven helpful in describing MRONJ-affected bone and changes in healthy bone during antiresorptive treatment [15,16,26,28].

According to the American Association of Oral and Maxillofacial Surgeons, radiographic imaging plays a crucial role in the assessment of MRONJ lesions. The reviewed literature further indicates that the diagnostic value of imaging may be enhanced by incorporating mathematically derived radiological markers [7].

A limitation of the present systematic review was that it analyzed publications which, although providing reliable evidence, offered predominantly exploratory findings suggesting that the fractal dimension may reflect microstructural bone changes in MRONJ. However, due to the limited sample sizes, substantial heterogeneity, and lack of standardization in fractal analysis methodologies, the results should be interpreted with caution. Larger, prospective, and methodologically standardized studies are required to confirm the clinical utility of fractal analysis in the diagnosis and monitoring of MRONJ. The key methodological aspects that should be considered in future research include standardized imaging protocols, consistent region of interest (ROI) selection, and uniform FD calculation methods.

The authors did not perform a meta-analysis due to the significant heterogeneity among the included studies, including differences in the study design, patient populations, treatment protocols, and reported outcomes. Because of this variability and the limited number of eligible studies, a quantitative synthesis was not considered methodologically appropriate.

## 5. Conclusions

The utilization of fractal dimension analysis has significantly increased in the field of dentistry. Although still under active development, this technique’s versatility, accessibility, and capacity to describe complex biological structures make it a promising tool for both research and clinical practice. The reviewed studies concluded that the fractal dimension decreases significantly for MRONJ-affected bones. FD analysis has the potential to become a viable and easily accessible method for quantitative bone assessment in MRONJ; however, due to its novelty and the inconsistency in findings and methodologies, further research is needed to validate, calibrate, and standardize these techniques. Until then, there remains no consensus on a standardized protocol for FD calculation in oral radiology. Therefore, the FD cannot be used as a single methodology for staging or diagnosis.

After further analysis of the correlations of other radiomorphometric markers with clinical and radiological findings, FA could be used in creating systems for the comprehensive analysis of bone structure.

## Figures and Tables

**Table 1:** Classification of MRONJ according to AAOMS.

Category/Stage	Clinical Description
At-risk	No clinically exposed necrotic bone; the patient is currently or has previously been treated with antiresorptive or antiangiogenic medications.
Stage 0	No clinically visible necrotic bone, but nonspecific symptoms (e.g., pain, sensory disturbances) or radiographic findings (e.g., sclerosis) may be present.
Stage 1	Exposed bone or a fistula that probes to bone in a patient with no signs of infection or inflammation.
Stage 2	Exposed bone or a fistula that probes to bone accompanied by signs of infection (pain, erythema, and possibly purulent drainage).
Stage 3	Exposed bone or fistula and infection and at least one of the following: pathologic fracture, extraoral fistula (e.g., cutaneous), oroantral/oronasal communication, or osteolysis extending beyond the alveolar bone (e.g., to the inferior border of the mandible or the sinus floor).

**Table 2:** Research articles included in the review.

No	Author	Number of Patients	Type of Image	Medication and Drug Administration	Results
1	Torres et al. (2011) [10]	36	CBCT	BP (no information about doses)	The region above the metal foramen shows the most promise for the detection of differences in the FD associated with bisphosphonates. The FD values of patients with BRONJ were significantly higher.
2	Sahin et al. (2019) [11]	66	OPG	*i.v.* and *p.o*.; BP, Mo (no information about doses)	Patients with more advanced MRONJ had higher mean FD values compared with those at stage 0, but the differences were significant only in 1 out of 4 selected ROIs—superior to the inferior alveolar canal (IAC), on the distal side of the mental foramen.
3	Bachtler et al. (2020) [12]	77	CBCT	BP and denosumab	The FD values of the group affected by MRONJ (stage 3) were significantly lower than in the control group.
4	Panneer-Selvam et al. (2022) [14]	18	OPG	*i.v.*; BP, Mo (no information about doses)	A comparison between the FD values of patients pre- and post-drug holiday showed no significant difference.
5	Aslan et al. (2024) [15]	24	OPG, CBCT	*i.v.* and *p.o*.; BP (no information about doses)	FA may be used to detect MRONJ-affected trabecular bone on PR but not on CBCT images.
6	Schulz RE et al. (2025) [16]	11	OPG	BP, Mo (no information about doses)	The FD can be used to differentiate healthy and necrotic bone. The values were the lowest for the necrotic sites, and for perinecrotic bone, the FD was slightly higher than the values of the non-affected bone.

Abbreviations: BP—bisphosphonate; BRONJ—bisphosphonate-related osteonecrosis of the jaw; CBCT—cone-beam computed tomography; FA—fractal analysis; FD—fractal dimension; IAC—inferior alveolar canal; *i.v.*—intravenous intake; Mo—monoclonal antibody (incl. Denosumab); MRONJ—medication-related osteonecrosis of the jaw; OPG—orthopantomography; *p.o.*—oral intake; ROI—region of interest.

**Table 3:** Comparison of FD values for ROI containing cancellous bone above the mandibular canal.

Author	Torres et al. (2011) [10]	Sahin et al. (2019) [11]	Bachtler et al. (2020) [12]	Aslan et al. (2024) [15]
Image modality	CBCT	OPG	CBCT	OPG
FD for MRONJ patients	1.698 ± 0.025	1.49 ± 0.036 * 1.56 ± 0.042 **	1.691 ± 0.0459	1.41 ± 0.04
FD for healthy bone (control)	1.670 ± 0.046		1.747 ± 0.0246	1.44 ± 0.02
*p* value	0.03	0.039	<0.001	0.007

* Patients without bone exposure (stage 0). ** Patients with bone exposure (stage 1, 2, 3, 4). Abbreviations: CBCT—cone-beam computed tomography; FD—fractal dimension; MRONJ—medication-related osteonecrosis of the jaw; OPG—orthopantomography; ROI—region of interest.

**Table 4:** Risk-of-bias evaluation using the Newcastle–Ottawa scale for quality assessment.

No	Reference	Sample Selection				Comparability		Exposure/Outcome		Total
		Adequate Case Definition	Representativeness of the Cases	Selection of Control	Definition of Control	Comparability of Cases	Controls Based on the Analysis	Ascertainment of Exposure	Data Completeness	
1	Torres et al. (2011) [10]	★	★	★	★	★	-	★	★	7
2	Sahin et al. (2019) [11]	★	★	★	★	★	★	★	★	8
3	Bachtler et al. (2020) [12]	★	★	-	-	★	★	★	★	6
4	Panneer-Selvam et al. (2022) [14]	★	-	-	-	★	-	★	★	4
5	Aslan et al. (2024) [15]	★	★	★	★	★	★	★	★	8
6	Schulz et al. (2025) [16]	★	★	★	★	★	★	★	★	8

Star (**★**) = item present; - = item not present.

## Data Availability

No new data were created or analyzed in this study. Data sharing is not applicable to this article.

## Supplementary Materials

The following supporting information can be downloaded at: https://www.mdpi.com/article/10.3390/dj14040207/s1, Table S1: PRISMA 2020 checklist.

## Abbreviations

AAOMS	American Association of Oral and Maxillofacial Surgeons
AD	Antiresorptive drug
ALP	Alkaline phosphatase
BA/TA	Bone area fraction
BP	Bisphosphonate
BRONJ	Bisphosphonate-related osteonecrosis of the jaw
CBCT	Cone-beam computed tomography
FA	Fractal analysis
FD	Fractal dimension
IAC	Inferior alveolar canal
*i.v.*	Intravenous intake
MCT	Mandibular cortical thickness
MGV	Mean grey value
Mo	Monoclonal antibody
MRONJ	Medication-related osteonecrosis of the jaw
NOS	Newcastle–Ottawa Scale
ONJ	Osteonecrosis of the jaw
OPG	Orthopantomography
*p.o.*	Oral intake
ROI	Region of interest
Tb.Sp	Trabecular separation
SDgv	Standard deviation grey value
